# Efficacy and safety of upadacitinib in patients with ankylosing spondylitis refractory to biologic therapy: 1-year results from the open-label extension of a phase III study

**DOI:** 10.1186/s13075-023-03128-1

**Published:** 2023-09-18

**Authors:** Xenofon Baraliakos, Désirée van der Heijde, Joachim Sieper, Robert D. Inman, Hideto Kameda, Yihan Li, Xianwei Bu, Anna Shmagel, Peter Wung, In-Ho Song, Atul Deodhar

**Affiliations:** 1 Rheumazentrum Ruhrgebiet Herne, Ruhr-University Bochum, Herne, Germany; 2 Rheumatology, Leiden University Medical Center, Leiden, The Netherlands; 3 Gastroenterology, Infectious Diseases and Rheumatology, Charité Universitätsmedizin Berlin, Berlin, Germany; 4 Schroeder Arthritis Institute, University Health Network, and University of Toronto, Toronto, ON Canada; 5 Internal Medicine, Toho University, Tokyo, Japan; 6 AbbVie Inc, North Chicago, IL USA; 7 Division of Arthritis & Rheumatic Diseases, Oregon Health & Science University, Portland, OR USA

**Keywords:** Ankylosing spondylitis, Janus kinase inhibitor, Open-label extension, Upadacitinib, Biologic, Tumor necrosis factor, Inadequate response, Refractory

## Abstract

**Background:**

Upadacitinib, a Janus kinase inhibitor, has demonstrated efficacy and an acceptable safety profile in patients with ankylosing spondylitis (AS) in the phase III SELECT-AXIS programs. We report the 1-year efficacy and safety in patients with AS and an inadequate response to biologic disease-modifying antirheumatic drugs (bDMARD-IR) from the SELECT-AXIS 2 study.

**Methods:**

Patients ≥ 18 years with active AS who met the modified New York criteria for AS and were bDMARD-IR received double-blind upadacitinib 15 mg once daily (QD) or placebo for 14 weeks. Patients who completed 14 weeks could enter an open-label extension and receive upadacitinib 15 mg QD for up to 2 years. Efficacy endpoints included the percentage of patients achieving ≥ 40% improvement in Assessment of SpondyloArthritis international Society response (ASAS40), Ankylosing Spondylitis Disease Activity Score (ASDAS) low disease activity (LDA), and ASDAS inactive disease (ID); and change from baseline in total and nocturnal back pain, and Bath Ankylosing Spondylitis Functional Index (BASFI). Subgroup analyses (bDMARD lack of efficacy versus intolerance, and prior tumor necrosis factor inhibitor [TNFi] versus interleukin-17 inhibitor [IL-17i] exposure) were conducted. Binary and continuous efficacy endpoints were assessed using non-responder imputation with multiple imputation (NRI-MI) and as observed (AO) analyses; and mixed-effects model repeated measures (MMRM) and AO, respectively. Safety was assessed based on adverse events. Data through week 52 are reported.

**Results:**

Of 420 randomized patients, 366 (continuous upadacitinib: *n* = 181; placebo to upadacitinib: *n* = 185) completed 52 weeks of treatment. At week 52, in the continuous upadacitinib and placebo to upadacitinib groups, ASAS40, ASDAS LDA, and ASDAS ID were achieved by 66% and 65%, 57% and 55%, and 26% and 25% (all NRI-MI); and change from baseline in total back pain, nocturnal back pain, and BASFI was -4.5 and -4.3, -4.6 and -4.4, and -3.6 and -3.5 (all MMRM), respectively. No new safety risks were identified. Subgroup analyses were consistent with the overall study population.

**Conclusions:**

Upadacitinib 15 mg QD demonstrated sustained improvement up to 52 weeks in bDMARD-IR patients with AS. Efficacy was generally similar in patients with lack of efficacy versus intolerance to bDMARDs and prior TNFi versus IL-17i exposure.

**Trial registration:**

NCT02049138.

**Supplementary Information:**

The online version contains supplementary material available at 10.1186/s13075-023-03128-1.

## Background

Axial spondyloarthritis (axSpA), a chronic, inflammatory rheumatic disease affecting the axial skeleton, is associated with back pain, spinal stiffness, peripheral articular and extra-musculoskeletal manifestations, and reduced quality of life [1–3]. The two main subgroups of axSpA are non-radiographic axSpA (nr-axSpA) and radiographic axSpA (r-axSpA), also known as ankylosing spondylitis (AS), in which definitive radiographic damage to the sacroiliac joints has already developed [2, 4]. The term r-axSpA has recently been introduced to reflect the fact that nr-axSpA and r-axSpA/AS are part of the same disease spectrum and are similar in terms of symptoms, disease burden, comorbidities, and treatment approach [4, 5]. AS and r-axSpA are synonymous within a clinical setting, and the term AS is retained in this manuscript for consistency with the study protocol and previously published data.

Treatment with a biologic disease-modifying antirheumatic drug (bDMARD), such as a tumor necrosis factor inhibitor (TNFi) or an interleukin-17 inhibitor (IL-17i), is recommended in patients with AS who have persistently high disease activity despite treatment with non-steroidal anti-inflammatory drugs (NSAIDs) [5, 6]. Many patients, however, do not achieve adequate response with their first bDMARD [7–10]. Recently, treatment with a Janus kinase (JAK) inhibitor was added to the 2022 Assessment of SpondyloArthritis international Society (ASAS)–European Alliance of Associations for Rheumatology (EULAR) recommendations as an option for patients who have intolerance or inadequate response (IR) to NSAIDs [5]. Recommendations also advise that patients who have an IR to their first bDMARD should switch to another bDMARD or a JAK inhibitor [5, 6].

The efficacy and safety of upadacitinib, an oral JAK inhibitor, has been evaluated in patients with axSpA in SELECT-AXIS 1 and SELECT-AXIS 2. In SELECT-AXIS 1, sustained efficacy over 2 years was observed with upadacitinib 15 mg once daily (QD) in bDMARD-naïve patients with active AS and an IR to NSAIDs, with a safety profile consistent with previous upadacitinib studies in rheumatoid arthritis and psoriatic arthritis [11–16]. The phase III, randomized, placebo-controlled, double-blind SELECT-AXIS 2 master protocol (NCT04169373) consists of two standalone studies in axSpA: a study in patients with nr-axSpA and another study in patients with AS and an IR to bDMARDs. The primary results from the bDMARD-IR AS study showed a significantly greater proportion of patients in the upadacitinib 15 mg QD group achieving the primary endpoint of ≥ 40% improvement in ASAS response (ASAS40) versus placebo at week 14 (45% vs 18%; *p* < 0.0001), with statistically significant improvements observed with upadacitinib versus placebo for all multiplicity-controlled secondary endpoints, including disease activity, inflammation by magnetic resonance imaging, pain, function, quality of life, spinal mobility, and enthesitis [17]. Rates of adverse events (AEs) were generally similar between treatment groups (except for more frequent serious AEs, infections, hepatic disorders, and neutropenia occurring with upadacitinib 15 mg QD compared with placebo) and there were no malignancies, major adverse cardiovascular events (MACEs), venous thromboembolic events (VTEs), or deaths reported with upadacitinib.

We report here the 1-year efficacy and safety from the SELECT-AXIS 2 bDMARD-IR AS study.

## Materials and methods

### Study design

Methods have been previously reported [17]. Briefly, patients in the initial double-blind period were randomized 1:1 to receive either oral upadacitinib 15 mg QD or placebo for 14 weeks. Patients from either treatment group who completed this double-blind period were eligible to enter an open-label extension and receive upadacitinib 15 mg QD for an additional 90 weeks, for a total treatment duration of 2 years. Here we report data through 52 weeks of the study.

### Patients

The study enrolled adult patients (aged ≥ 18 years) with AS who met the modified New York criteria based on central reading of sacroiliac joint radiographs and had active disease, defined as a Bath Ankylosing Spondylitis Disease Activity Index (BASDAI) score of ≥ 4 and a patient’s assessment of total back pain score of ≥ 4 (numeric rating scale 0–10). Patients had to have an IR to ≥ 2 NSAIDs and an IR to bDMARDs, defined as either a lack of efficacy (after ≥ 12 weeks of treatment at an adequate dose as assessed by the investigator) or intolerance (regardless of treatment duration). Prior exposure to two bDMARDs (lack of efficacy to one bDMARD and intolerance to the other, but not lack of efficacy to both bDMARDs) was allowed for ≤ 30% of patients. Patients receiving stable doses of concomitant oral corticosteroids, NSAIDs, and conventional synthetic disease-modifying antirheumatic drugs at baseline were eligible. Prior exposure to a JAK inhibitor was not permitted.

### Efficacy endpoints

The following endpoints were assessed through week 52: the percentage of patients achieving ASAS40 response, AS Disease Activity Score (ASDAS) using C-reactive protein low disease activity (LDA; < 2.1), ASDAS inactive disease (ID; < 1.3), ≥ 50% improvement in BASDAI (BASDAI50), ≥ 20% improvement in ASAS response (ASAS20), ASAS partial remission (PR; absolute score of ≤ 2 units for each of the four domains of ASAS40); change from baseline in linear Bath Ankylosing Spondylitis Metrology Index (BASMI), Maastricht Ankylosing Spondylitis Enthesitis Score (MASES), ASAS Health Index (HI), Ankylosing Spondylitis Quality of Life (ASQoL), patient’s assessment of total back pain and nocturnal back pain (numeric rating scale 0–10), and Bath Ankylosing Spondylitis Functional Index (BASFI). Additional efficacy endpoints are listed in Supplementary Table 2.

### Safety endpoints

Safety was assessed by treatment-emergent AEs (TEAEs), serious AEs, AEs leading to discontinuation, prespecified AEs of special interest, and laboratory parameters. TEAEs were defined as AEs with an onset date on or after the first dose of study drug and up to 30 days after the last dose of study drug. The toxicity grading scale is based on National Cancer Institute Common Toxicity Criteria for Adverse Events version 4.03.

### Statistical analysis

Efficacy analyses were performed at 52 weeks on all randomized patients who received ≥ 1 dose of study drug. No statistical comparisons were performed between the treatment groups (continuous upadacitinib versus placebo to upadacitinib).

For binary efficacy endpoints, non-responder imputation with multiple imputation (NRI-MI) and as observed (AO) analyses are presented. In the NRI-MI analysis, patients who prematurely discontinued study drug (including those who did not enter open-label extension) or were rescued were considered non-responders; MI was used to handle missing data due to COVID-19.

For continuous efficacy endpoints, estimated change from baseline from mixed-effects model repeated measures (MMRM) and AO data are reported. MMRM included the categorical fixed effects of treatment, visit, and treatment-by-visit interaction as fixed factors and baseline value as covariate, and stratification factor of screening high-sensitivity C-reactive protein level (≤ upper limit of normal [ULN] vs > ULN).

To better understand the efficacy of upadacitinib in clinically relevant subgroups, post hoc analyses for efficacy endpoints at week 52 were performed in subgroups of patients who had discontinued a prior bDMARD due to lack of efficacy versus intolerance, and patients with prior TNFi exposure versus prior IL-17i exposure, respectively.

The safety analysis set included all patients who received ≥ 1 dose of upadacitinib 15 mg QD at any point in the study. Safety outcomes were assessed up to the cut-off date of May 19, 2022. Exposure-adjusted event rates (events per 100 patient-years [E/100 PY]) are reported.

## Results

### Patient disposition and baseline characteristics

**Fig. 1:**
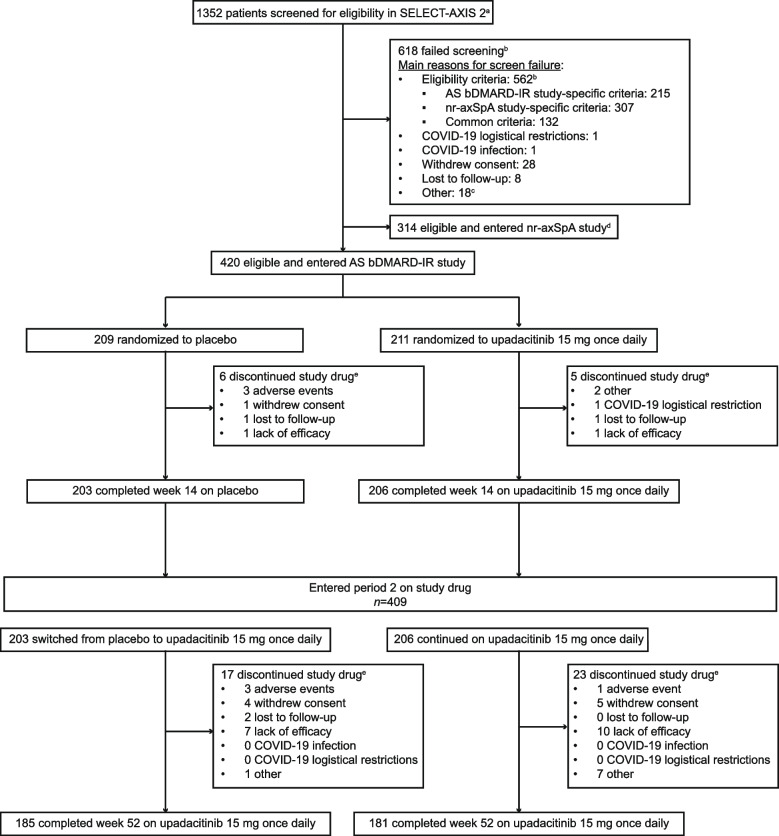
Patient disposition through week 52. ^a^Patients were screened between November 26, 2019 and May 20, 2021 for the SELECT-AXIS 2 master protocol, which used a common screening platform to assign patients to either the AS bDMARD-IR study or the nr-axSpA study. ^b^Patients could have multiple criteria or multiple reasons for screening failure. ^c^Other reasons included imaging, site, or system issues. ^d^Patients did not fail screening. ^e^Primary reason for discontinuation provided. *AS* ankylosing spondylitis, *bDMARD* biologic disease-modifying antirheumatic drug, *IR* inadequate response, *nr-axSpA* non-radiographic axial spondyloarthritis

Of the 420 patients randomized in the double-blind treatment period, 409 entered the open-label extension phase at week 14 (continuous upadacitinib: *n* = 206; placebo to upadacitinib: *n* = 203). Of these, 181 (88%) and 185 (91%) patients in the continuous upadacitinib and placebo to upadacitinib groups, respectively, completed 52 weeks of treatment (Fig. 1).

Patient demographics and baseline disease characteristics have been reported previously [17]. Treatment arms were generally well balanced and reflective of an active AS bDMARD-IR population in both arms. Among this bDMARD-IR population, the majority of patients failed their prior bDMARD(s) due to lack of efficacy. In addition, the majority of patients had been exposed to TNFis, with around 20% having prior IL-17i exposure (Supplementary Table 1).

### Efficacy

**Fig. 2:**
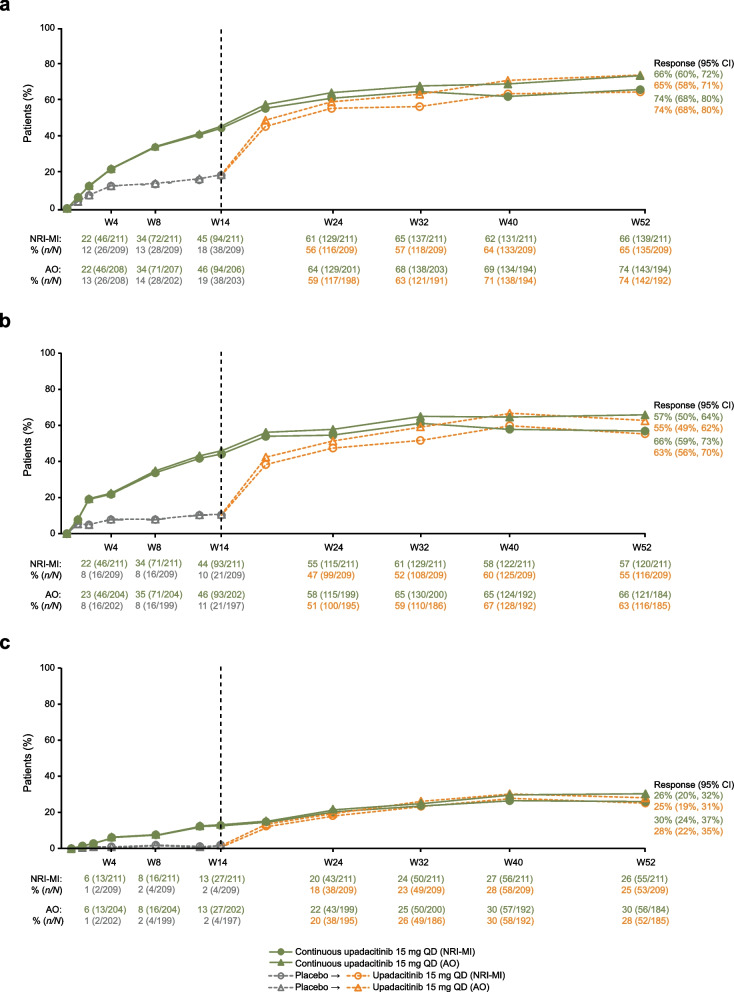
ASAS40 (**a**), ASDAS LDA (**b**), and ASDAS ID (**c**) responses over time. Patients initially randomized to receive placebo received open-label upadacitinib beginning at week 14. NRI-MI (NRI incorporating MI to handle missing data due to COVID-19) and AO analyses were used. *AO* as observed, *ASAS40* ≥ 40% improvement in Assessment of SpondyloArthritis international Society response, *ASDAS* Ankylosing Spondylitis Disease Activity Score, *CI* confidence interval, *ID* inactive disease, *LDA* low disease activity, *MI* multiple imputation, *NRI* non-responder imputation, *QD* once daily, *W* week

The percentage of patients who achieved the primary efficacy endpoint of ASAS40 response at week 14 (NRI-MI: 45%) continued to increase with continuous upadacitinib treatment through 52 weeks (NRI-MI: 66%; Fig. 2A). Patients who switched from placebo to upadacitinib at week 14 showed a rapid initial response and reached similar ASAS40 response rates to those of the continuous upadacitinib group between weeks 32 and 40, which was maintained through week 52 (NRI-MI: 65%).

The percentage of patients who achieved ASDAS LDA and ID generally followed a similar pattern, with continued improvements through week 52 for both the continuous upadacitinib group (NRI-MI: 57% and 26%, respectively) and the placebo to upadacitinib group (NRI-MI: 55% and 25%, respectively; Fig. 2B and 2C). Other disease activity outcomes, including ASAS20 response, ASAS PR, and BASDAI50, followed similar trends (Supplementary Fig. 1). Response rates based on AO analyses were numerically higher than NRI-MI but followed a similar trend as noted for ASAS40, ASDAS LDA, and ASDAS ID (Fig. 2). Similar trends were also observed at week 52 in rates of ASDAS major improvement and ASDAS clinically important improvement in the continuous upadacitinib and placebo to upadacitinib groups, respectively (Supplementary Table 2).

**Fig. 3:**
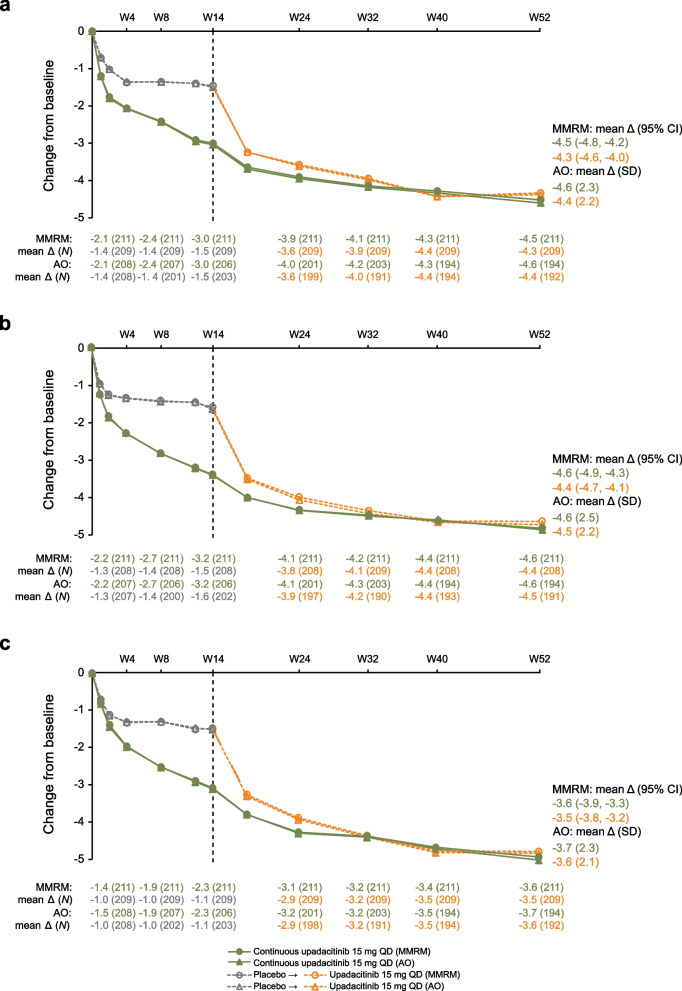
Mean change in total back pain (**a**), nocturnal back pain (**b**), and BASFI (**c**) over time. Patients initially randomized to receive placebo received open-label upadacitinib beginning at week 14. MMRM and AO analyses were used. *Δ* change, *AO* as observed, *BASFI* Bath Ankylosing Spondylitis Functional Index, *CI* confidence interval, *MMRM* mixed-effects model repeated measures, *QD* once daily, *SD* standard deviation, *W* week

Improvements observed at week 14 were maintained in the continuous upadacitinib group through week 52, and similar improvements were achieved in the placebo to upadacitinib group at week 52, for change from baseline in total back pain, nocturnal back pain, and function, respectively (Fig. 3A–C). Similarly, improvements in mobility (BASMI), enthesitis (MASES), and quality of life (ASQoL and ASAS HI) were observed in both treatment groups (Supplementary Fig. 2). AO results followed a similar trend to the MMRM results for these endpoints. Improvements were also observed in duration and severity of morning stiffness, patient global assessment of pain, patient global assessment of disease activity, and Functional Assessment of Chronic Illness Therapy—Fatigue (FACIT-F) (Supplementary Table 2).

In the subgroups of patients who had discontinued prior bDMARD treatment due to lack of efficacy versus intolerance, as well as the subgroups of patients who had prior treatment with TNFis versus IL-17is, similar response rates were observed for ASAS40 and other endpoints at week 52 in both the continuous upadacitinib and placebo to upadacitinib groups (Supplementary Tables 3 and 4). These results were consistent with the overall study population.

### Safety

Safety was assessed through the cut-off date of May 19, 2022 in 414 patients (534.4 PY) who received ≥ 1 dose of upadacitinib 15 mg QD. The median exposure to upadacitinib was 487 days and 330 (80%) patients had ≥ 12 months’ exposure.

Overall, the rate of TEAEs was 164.1 E/100 PY, with the most common being COVID-19, nasopharyngitis, and upper respiratory tract infection (Table 1). The rates of serious AEs and AEs leading to discontinuation of study drug were 9.9 E/100 PY and 3.0 E/100 PY, respectively, and there was one death due to polytrauma (Table 1). No pattern of AEs leading to discontinuation was observed. The most common serious AEs were COVID-19 pneumonia and COVID-19. None of the serious COVID-19 events led to discontinuation of study drug.

**Table 1:** Treatment-emergent AEs

Exposure-adjusted event rates, E (E/100 PY)	Any upadacitinib 15 mg QD^a^ (*n* = 414; PY = 534.4)
Any AE	877 (164.1)
Serious AE	53 (9.9)
AE leading to discontinuation of study drug	16 (3.0)
Any COVID-19-related AE	81 (15.2)
Any death^b^	1 (0.2)
Infection	301 (56.3)
Serious infection	24 (4.5)
Opportunistic infection^c^	0
Herpes zoster^d^	19 (3.6)
Active tuberculosis	0
Malignancy other than NMSC^e^	1 (0.2)
NMSC	2 (0.4)
Adjudicated MACE^f^	1 (0.2)
Adjudicated VTE^g^	2 (0.4)
Adjudicated gastrointestinal perforation	0
Renal dysfunction^h^	1 (0.2)
Anemia	11 (2.1)
Lymphopenia	3 (0.6)
Neutropenia	19 (3.6)
Hepatic disorder	47 (8.8)
Uveitis^i^	7 (1.3)
Inflammatory bowel disease^j^	1 (0.2)
Psoriasis	2 (0.4)

^a^All patients who received ≥ 1 dose of upadacitinib 15 mg QD.

^b^One patient died due to polytrauma.

^c^Excluding tuberculosis and herpes zoster.

^d^One serious AE of herpes zoster.

^e^One severe AE of colon neoplasm with liver metastases.

^f^MACE defined as cardiovascular death (includes acute myocardial infarction, sudden cardiac death, heart failure, cardiovascular procedure-related death, death due to cardiovascular hemorrhage, fatal stroke, pulmonary embolism, and other cardiovascular causes), non-fatal myocardial infarction, and non-fatal stroke. One AE of basal ganglia hemorrhage.

^g^VTE included fatal and non-fatal deep vein thrombosis and pulmonary embolism. One serious AE of pulmonary embolism. One non-serious AE of deep vein thrombosis; study drug was discontinued.

^h^One serious AE of acute kidney injury; study drug dose remained unchanged.

^i^One serious event of recurrent uveitis and one serious event of iridocyclitis in a patient with a history of uveitis. One new serious AE of uveitis. One new serious AE of iridocyclitis.

^j^One grade 2 event of Crohn’s disease.

*AE* adverse event, *E* events, *MACE* major adverse cardiovascular event, *NMSC* non-melanoma skin cancer, *PY* patient-years, *QD* once daily, *VTE* venous thromboembolic event

Serious infection and herpes zoster rates were 4.5 E/100 PY and 3.6 E/100 PY, respectively, and there were no cases of opportunistic infection or active tuberculosis. Fourteen of the 24 reported serious infections were COVID-19 events. None of the treatment-emergent serious infections led to discontinuation of study drug. The case of disseminated herpes zoster occurred in a 62-year-old Japanese male with risk factors (history of diabetes and chicken pox, and not receiving the herpes zoster vaccination) during the open-label extension period, which resulted in the patient requiring hospitalization; upadacitinib treatment was interrupted and restarted upon improvement. There was also one non-serious event of post-herpetic neuralgia, which led to treatment discontinuation. All other herpes zoster events were of mild or moderate severity, non-serious, and involved one dermatome only.

There was one case of colon cancer with metastases to the liver in a 58-year-old patient with a 20-year history of smoking one pack of cigarettes per day. The patient discontinued upadacitinib treatment. Two cases of non-melanoma skin cancer, both of basal cell carcinoma, occurred on sun-exposed areas of the skin; one was a relapse in a patient with prior history of cutaneous basal cell carcinoma.

One case of adjudicated MACE occurred, which was a serious, non-fatal hemorrhagic stroke in a 47-year-old male patient with a history of smoking. Upadacitinib treatment was temporarily interrupted. Two VTEs were reported: one deep vein thrombosis occurring in a 59-year-old white male with risk factors including obesity, age > 40 years, and prolonged immobilization due to hip pain; and one pulmonary embolism occurring in a 30-year-old white female with risk factors including oral contraceptive use, sedentary lifestyle, and being overweight. Study drug was discontinued in both cases.

The rate of hepatic disorders was 8.8 E/100 PY, with the majority of events being mild alanine aminotransferase or aspartate aminotransferase elevations. There were no cases of Hy’s law observed [18]. One case of acute kidney injury was reported in a 52-year-old Asian male. The event was serious, but study drug dose remained unchanged.

There were seven events of uveitis. One patient with a history of uveitis had one event of recurrent uveitis during double-blind treatment and one event of iridocyclitis during open-label treatment; both events were serious. One patient had a serious AE of uveitis occurring during open-label treatment, which led to interruption of study drug. One patient had a serious AE of iridocyclitis during open-label treatment, but study drug dose remained unchanged. All of the other events were considered mild or moderate. None of these events resulted in permanent study drug discontinuation.

One case of inflammatory bowel disease was reported in a patient newly diagnosed with Crohn’s disease, and without a history of inflammatory bowel disease, on day 7 of receiving upadacitinib in the double-blind period.

Mean levels of hemoglobin, lymphocytes, and neutrophils remained relatively stable over 52 weeks, with grade 3 changes reported in 1, 3, and 7 patients, respectively (Supplementary Table 5).

## Discussion

This study reported the efficacy and safety of upadacitinib 15 mg QD in patients with AS who had an IR to bDMARDs (including TNFi or IL-17i therapy) through 52 weeks. The responses that were observed in the initial double-blind period of the study [17] further improved after week 14 and were sustained through 52 weeks in the open-label extension. These 1-year data demonstrated consistent improvement and maintenance of response with upadacitinib treatment in treatment-refractory patients with AS across a wide range of clinically relevant domains encompassing disease activity, pain, function, enthesitis, and quality of life, whether using NRI-MI/MMRM analyses or AO. In addition, patients who were randomized to placebo and then switched to upadacitinib at week 14 showed a rapid response, followed by a similar magnitude of response compared with the continuous upadacitinib group at week 52.

The results of this study are in line with other studies where maintenance of efficacy over 1 year or longer has been observed in patients with AS treated with TNFi or IL-17i therapy, or another JAK inhibitor [10, 19–21]. Notably, most other studies were conducted in mixed bDMARD-naïve and bDMARD-IR populations, whereas our study was conducted entirely in bDMARD-IR patients. Compared with the only other bDMARD-IR phase III trial in which the entire AS population had prior exposure to a bDMARD (a study of ixekizumab in patients with an IR to TNFis), response rates for ASAS40 at week 52 and other endpoints such as ASDAS LDA were numerically higher in the upadacitinib trial compared with the ixekizumab trial (ASAS40 at week 52: 66% for upadacitinib vs 34% for ixekizumab) [21]. However, this was not a head-to-head comparison and should be interpreted with caution.

The efficacy outcomes in this 1-year analysis in bDMARD-IR patients with AS are also consistent with previous reports for the SELECT-AXIS 1 study in bDMARD-naïve patients with AS and NSAID-IR [12]. Overall, similar proportions of patients treated with upadacitinib 15 mg achieved clinical efficacy outcomes in this analysis of bDMARD-IR patients with AS compared with the SELECT-AXIS 1 study in bDMARD-naïve patients with AS and NSAID-IR at week 52 [12]. It should be noted, however, that cross-study comparisons should be interpreted with caution.

Efficacy outcomes at week 52 were generally similar across subgroups of patients who had discontinued prior bDMARD treatment due to lack of efficacy versus intolerance, and prior TNFi versus IL-17i exposure, and were consistent with the overall bDMARD-IR population reported in this study. Numerically lower responses were observed for some endpoints in the prior IL-17i exposure subgroup, but this subgroup had a limited number of patients so additional studies would be needed to further evaluate this.

Treatment with upadacitinib was generally well tolerated throughout the study. No new safety risks were identified compared with the known safety profile of upadacitinib [12, 17]. Rates of serious infections, herpes zoster, and neutropenia were reported at ≤ 5.0 E/100 PY, and malignancies, MACEs, and VTEs were uncommon in this population (0.2, 0.2, and 0.4 E/100 PY, respectively). These rates are numerically lower than those seen in 1-year studies of upadacitinib 15 mg QD in patients with rheumatoid arthritis and psoriatic arthritis; however, those studies included older patient populations [11, 13]. There were no cases of opportunistic infection or active tuberculosis. Rates of AEs leading to discontinuation of study drug were also low. Given the timing of the study during the early stages of the global pandemic, COVID-19 was the leading reason for study drug discontinuation (four cases of COVID-19, 10 cases of COVID-19 pneumonia, specifically).

The main limitations of this study are the lack of a comparator arm and the open-label nature of the study during the extension period. Defining a patient as having an IR due to a lack of efficacy or an intolerance to a bDMARD was based solely on the discretion of the investigators, although this is in line with the approach used in other studies [7, 22]. The lack of an established definition of IR may explain potential patient selection variability, which may have influenced the magnitude of treatment responses. The small patient numbers in some of the subgroups analyzed—in particular, the prior IL-17i exposure subgroup—was also a limitation. Although this analysis covered a period of 1 year, further results from this ongoing extension study will provide more data and determine whether the maintenance of response extends through 2 years, as observed in SELECT-AXIS 1 [15].

## Conclusions

Upadacitinib 15 mg QD demonstrated sustained improvement up to week 52 in bDMARD-IR patients with active AS during open-label treatment. Similar efficacy was observed at week 52 in patients on continuous upadacitinib and those who switched from placebo to upadacitinib 15 mg QD at week 14. Treatment with upadacitinib 15 mg QD was generally safe and well tolerated, with no new safety risks identified compared with the known safety profile of upadacitinib. The efficacy of upadacitinib 15 mg QD was generally similar across subgroups of patients who had discontinued prior bDMARD treatment due to lack of efficacy versus intolerance, and prior TNFi versus IL-17i exposure, and consistent with the overall SELECT-AXIS 2 bDMARD-IR AS population. These data suggest that upadacitinib is an effective treatment for bDMARD-IR patients with AS up to 1 year of treatment.

### Supplementary Information

**Additional file 1.**

## Data Availability

AbbVie is committed to responsible data sharing regarding the clinical trials we sponsor. This includes access to anonymized, individual, and trial-level data (analysis datasets), as well as other information (e.g., protocols and clinical study reports), provided the trials are not part of an ongoing or planned regulatory submission. This includes requests for clinical trial data for unlicensed products and indications. These clinical trial data can be requested by any qualified researchers who engage in rigorous, independent scientific research, and will be provided following review and approval of a research proposal and statistical analysis plan, and execution of a Data Sharing Agreement. Data requests can be submitted at any time and the data will be accessible for 12 months, with possible extensions considered. For more information on the process, or to submit a request, visit https://www.abbvie.com/our-science/clinical-trials/clinical-trials-data-and-information-sharing/data-and-information-sharing-with-qualified-researchers.html.

## Abbreviations

Δ: Change
AE: Adverse event
ALT: Alanine aminotransferase
AO: As observed
AS: Ankylosing spondylitis
ASAS: Assessment of SpondyloArthritis international Society
ASAS20: ≥ 20% Improvement in Assessment of SpondyloArthritis international Society response
ASAS40: ≥ 40% Improvement in Assessment of SpondyloArthritis international Society response
ASDAS: Ankylosing Spondylitis Disease Activity Score
ASQoL: Ankylosing Spondylitis Quality of Life
axSpA: Axial spondyloarthritis
BASDAI: Bath Ankylosing Spondylitis Disease Activity Index
BASDAI50: ≥ 50% improvement in Bath Ankylosing Spondylitis Disease Activity Index
BASFI: Bath Ankylosing Spondylitis Functional Index
BASMI: Bath Ankylosing Spondylitis Metrology Index
bDMARD: Biologic disease-modifying antirheumatic drug
CI: Confidence interval
E: Events
FACIT-F: Functional Assessment of Chronic Illness Therapy—Fatigue
HI: Health Index
ID: Inactive disease
IL-17i: Interleukin-17 inhibitor
IR: Inadequate response
JAK: Janus kinase
LDA: Low disease activity
MACE: Major adverse cardiovascular event
MASES: Maastricht Ankylosing Spondylitis Enthesitis Score
MI: Multiple imputation
MMRM: Mixed-effects model repeated measures
NMSC: Non-melanoma skin cancer
nr-axSpA: Non-radiographic axial spondyloarthritis
NRI: Non-responder imputation
NSAID: Non-steroidal anti-inflammatory drug
PR: Partial remission
PY: Patient-years
QD: Once daily
r-axSpA: Radiographic axial spondyloarthritis
SD: Standard deviation
TNFi: Tumor necrosis factor inhibitor
ULN: Upper limit of normal
VTE: Venous thromboembolic event
W: Week
