# Supplementary material for: Efficacy and safety of upadacitinib in patients with ankylosing spondylitis refractory to biologic therapy: 1-year results from the open-label extension of a phase III study
Source: Arthritis Res Ther. 2023 Sep 18;25:172. doi: 10.1186/s13075-023-03128-1 (PMC10506267; doi:10.1186/s13075-023-03128-1)
Supplement: Supplementary file 1 — Additional file 1. [file 13075_2023_3128_MOESM1_ESM.docx]

# Supplementary appendix

**Supplementary Fig. 1** ASAS20 (a), ASAS PR (b), and BASDAI50 (c) responses over time


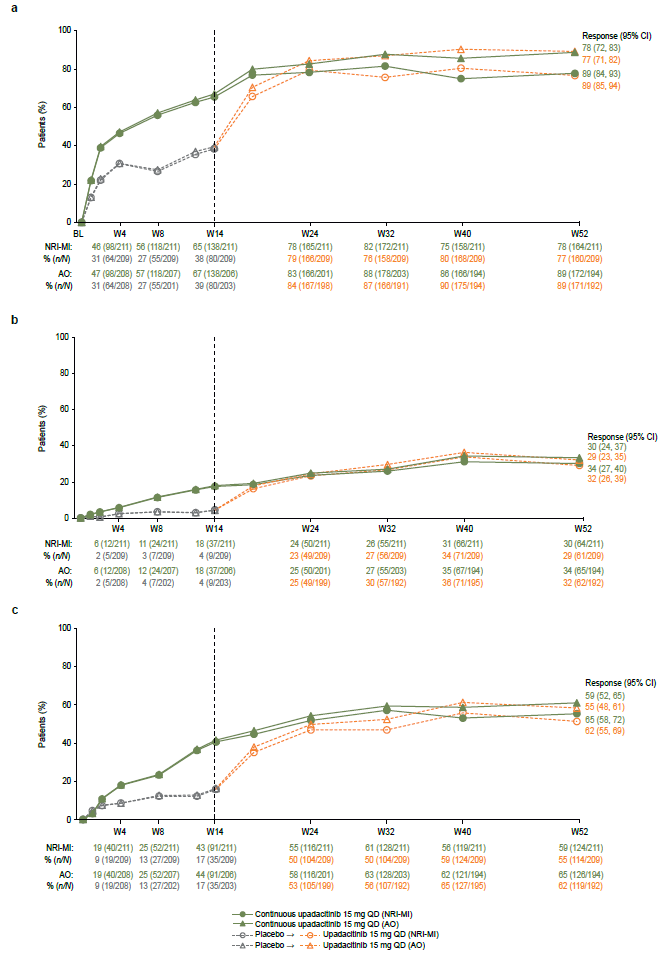


Patients initially randomized to receive placebo received open-label upadacitinib beginning at week 14. NRI-MI (NRI incorporating MI to handle missing data due to COVID-19) and AO analyses were used. *AO* as observed, *ASAS* Assessment of SpondyloArthritis international Society, *ASAS20* ≥ 20% improvement in Assessment of SpondyloArthritis international Society response, *BASDAI50* ≥ 50% improvement in Bath Ankylosing Spondylitis Disease Activity Index, *CI* confidence interval, *MI* multiple imputation, *NRI* non-responder imputation, *QD* once daily, *PR* partial remission, *W* week

**Supplementary Fig. 2** Mean change in BASMI (a), MASES (b), ASQoL (c), and ASAS HI (d) over time


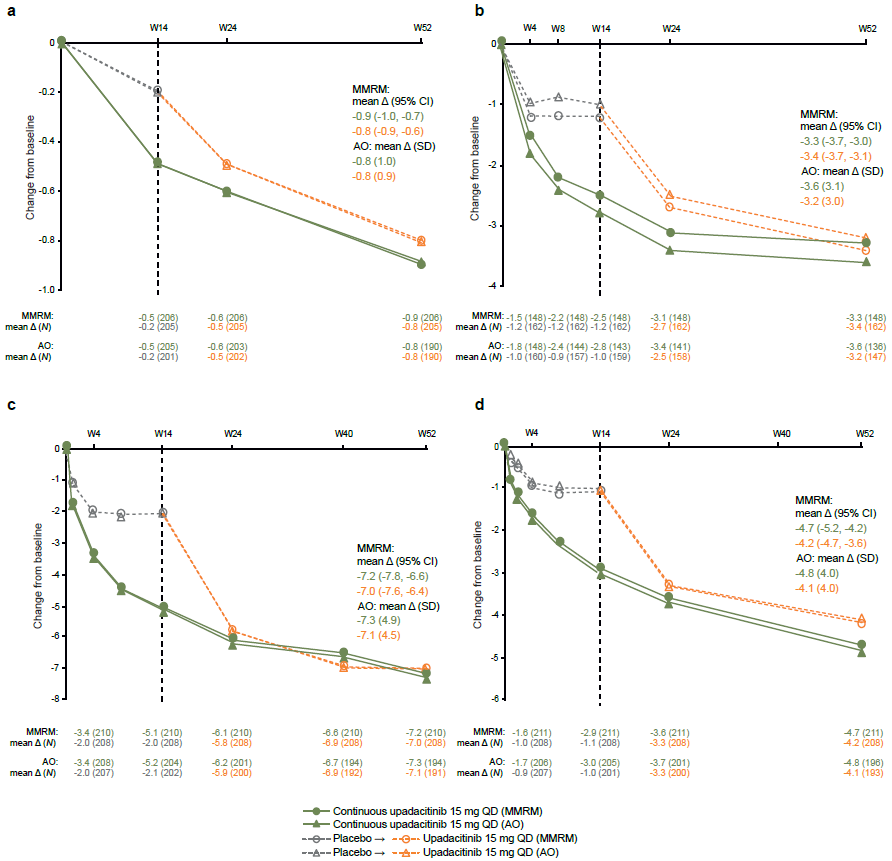


Patients initially randomized to receive placebo received open-label upadacitinib beginning at week 14. MMRM and AO analyses were used. *Δ* change, *AO* as observed, *ASAS* Assessment of SpondyloArthritis international Society, *ASQoL* Ankylosing Spondylitis Quality of Life, *BASMI* Bath Ankylosing Spondylitis Metrology Index, *CI* confidence interval, *HI* Health Index, *MASES* Maastricht Ankylosing Spondylitis Enthesitis Score, *MMRM* mixed-effects model repeated measures, *QD* once daily, *W* week

**Supplementary Table 1.** Number of patients in the post hoc subgroup analysis

| **Subgroup, n (%)** | **Placebo → upadacitinib 15 mg QD**  **(*n=*209)** | **Continuous upadacitinib  15 mg QD**  **(*n=*211)** |
| --- | --- | --- |
| bDMARD-IR (lack of efficacy)^a^ | 159 (76.1) | 166 (78.7) |
| bDMARD-IR (intolerance)^a^ | 66 (31.6) | 61 (28.9) |
| TNFi exposed^b^ | 183 (87.6) | 181 (85.8) |
| IL-17i exposed^b^ | 36 (17.2) | 38 (18.0) |

^a^Includes 33 patients with both lack of efficacy and intolerance to prior bDMARDs. ^b^Includes 19 patients with both prior TNFi and IL-17i exposure

*bDMARD* biologic disease-modifying antirheumatic drug, *IL-17i* interleukin-17 inhibitor, *IR*inadequate response, *QD* once daily, *TNFi* tumor necrosis factor inhibitor

**Supplementary Table 2.** Additional efficacy results at week 52

|  | Placebo → upadacitinib  15 mg QD | Continuous upadacitinib  15 mg QD |
| --- | --- | --- |
| Patients, % | NRI-MI / AO  *n=*209 / 185 | NRI-MI / AO *n=*211 / 184 |
| ASDAS major improvement | 44.9 / 49.7 | 45.3 / 52.7 |
| ASDAS CII | 66.9 / 77.3 | 69.0 / 79.9 |
| Mean change from baseline | MMRM / AO (SD)  *n=*209 / 192 | MMRM / AO (SD)  *n=*211 / 194 |
| Severity of morning stiffness^a^ | -4.55 / -4.66 (2.17) | -4.46 / -4.49 (2.54) |
| Duration of morning stiffness^a^ | -4.14 / -4.12 (2.51) | -3.97 / -4.10 (3.02) |
| Patient global assessment of pain^a^ | -4.37 / -4.46 (2.06) | -4.61 / -4.67 (2.32) |
| Patient global assessment of disease activity^a^ | -4.22 / -4.29 (2.20) | -4.54 / -4.62 (2.35) |
| Fatigue/tiredness^a^ | -3.57 / -3.60 (2.40) | -3.91 / -4.01 (2.45) |
| FACIT-F | 10.88^b^ / 10.78 (8.95)^c^ | 12.38^d^ / 12.55 (9.20)^e^ |
| Swollen joint count (of 66 joints) | -1.3^b^ / -1.4 (3.4)^f^ | -1.2^g^ / -1.1 (3.3)^f^ |
| Tender joint count (of 68 joints) | -3.3^b^ / -3.3 (6.7)^f^ | -3.3^g^ / -3.4 (6.1)^f^ |

^a^NRS score 0–10. ^b^*n=*205. ^c^*n=*188. ^d^*n=*209. ^e^*n=*191. ^f^*n=*190. ^g^*n=*206

*AO* as observed, *ASDAS* Ankylosing Spondylitis Disease Activity Score, *CII* clinically important improvement, *FACIT-F* Functional Assessment of Chronic Illness Therapy—Fatigue, *MI* multiple imputation, *MMRM* mixed-effects model repeated measures, *NRI* non-responder imputation, *NRS* numeric rating scale, *QD* once daily, *SD* standard deviation

**Supplementary Table 3.** Efficacy results at week 52 in bDMARD-IR subgroups

|  | **Lack of efficacy of bDMARDs**^a^ | | **Intolerance to bDMARDs**^a^ | |
| --- | --- | --- | --- | --- |
|  | **Placebo → upadacitinib 15 mg QD**  ***n=*159 /144** | **Continuous upadacitinib 15 mg QD**  ***n=*166 / 151** | **Placebo → upadacitinib 15 mg QD**  ***n=*66 / 61** | **Continuous upadacitinib 15 mg QD**  ***n=*61 / 58** |
| **Patients, %** | **NRI-MI / AO** | **NRI-MI / AO** | **NRI-MI / AO** | **NRI-MI / AO** |
| ASAS40 response | 64.2 / 74.3 | 65.1 / 74.2 | 60.6 / 70.5 | 68.9 / 72.4 |
| ASAS20 response | 76.1 / 90.3 | 75.3 / 87.4 | 74.2 / 85.2 | 83.6 / 89.7 |
| ASAS PR | 28.3 / 31.3 | 30.1 / 33.8 | 28.8 / 32.8 | 27.9 / 29.3 |
| BASDAI50 | 53.5 / 61.1 | 57.8 / 64.9 | 54.5 / 63.9 | 63.9 / 67.2 |
| ASDAS ID | 23.1 / 25.5^b^ | 24.4 / 29.4^c^ | 30.3 / 34.4 | 28.8 / 32.1^d^ |
| ASDAS LDA | 55.1 / 63.5^b^ | 56.2 / 66.4^c^ | 51.5 / 57.4 | 55.1 / 60.4^d^ |
| **Mean change from baseline^e^** | **MMRM / AO (SD)** | **MMRM / AO (SD)** | **MMRM / AO (SD)** | **MMRM / AO (SD)** |
| ASDAS | -1.92 / -1.92 (1.01)^b^ | -1.97 / -1.97 (1.04)^c^ | -1.92 / -1.96 (0.91) | -1.99 / -2.04 (1.11)^d^ |
| Total back pain^f^ | -4.41 / -4.45 (2.23) | -4.54 / -4.64 (2.32) | -4.04 / -4.16 (2.10) | -4.34 / -4.33 (2.27) |
| Nocturnal back pain^f^ | -4.48^g^ / -4.56 (2.23)^c^ | -4.56 / -4.59 (2.53) | -3.93 / -4.15 (2.09) | -4.38 / -4.36 (2.56) |
| Duration of morning stiffness^f^ | -4.24 / -4.22 (2.61) | -3.89 / -4.02 (3.18) | -3.89 / -3.92 (2.35) | -3.98 / -4.03 (2.82) |
| Severity of morning stiffness^f^ | -4.59 / -4.72 (2.28) | -4.43 / -4.44 (2.65) | -4.34 / -4.49 (1.83) | -4.39 / -4.43 (2.35) |
| Fatigue/tiredness^f^ | -3.67 / -3.67 (2.41) | -3.89 / -4.02 (2.53) | -3.34 / -3.43 (2.30) | -3.93 / -3.97 (2.43) |
| FACIT-F | 10.80^h^ / 10.56 (8.91)^i^ | 12.78^j^ / 13.16 (9.51)^k^ | 11.00^l^ / 11.23 (8.82)^m^ | 11.56 / 11.15 (7.32)^n^ |
| BASFI  ASQoL | -3.58 / -3.63 (2.12) | -3.59 / -3.65 (2.34) | -3.29 / -3.34 (1.85) | -3.67 / -3.70 (2.32) |
|  | -6.91^g^ / -6.95 (4.53)^c^ | -7.29^o^ / -7.49 (4.92) | -6.89 / -6.98 (4.18) | -6.98 / -7.07 (4.87) |
| ASAS HI | -4.15^g^ / -4.13 (4.02) | -4.81 / -4.95 (3.95)^p^ | -3.88 / -3.78 (3.85)^q^ | -4.43 / -4.64 (3.77) |
| BASMI | -0.72^r^ / -0.73 (0.87)^c^ | -0.84^s^ / -0.82 (1.03)^t^ | -0.85^l^ / -0.84 (0.77)^m^ | -0.78^u^ / -0.82 (1.04)^v^ |
| MASES | -3.5^w^ / -3.3 (3.1)^x^ | -3.5^y^ / -3.8 (3.2)^z^ | -3.2^aa^ / -3.0 (3.1)^bb^ | -3.1^cc^ / -3.4 (2.7)^dd^ |

^a^Includes 33 patients with both lack of efficacy and intolerance to prior bDMARDs. ^b^*n=*137. ^c^*n=*143. ^d^*n=*53. ^e^Increasing negative values indicate improvement, apart from FACIT-F in which increasing positive values indicate improvement. ^f^NRS score 0–10. ^g^*n=*158. ^h^*n=*156. ^i^*n=*141. ^j^*n=*164. ^k^*n=*149. ^l^*n=*65. ^m^*n=*60. ^n^*n=*57. ^o^*n=*165. ^p^*n=*153. ^q^*n=*62. ^r^*n=*155. ^s^*n=*162. ^t^*n=*147. ^u^*n=*59. ^v^*n=*56. ^w^*n=*124. ^x^*n=*111. ^y^*n=*117. ^z^*n=*107. ^aa^*n=*50. ^bb^*n=*46. ^cc^*n=*42. ^dd^*n=*39

*AO* as observed, *ASAS* Assessment of SpondyloArthritis international Society, *ASAS20* ≥ 20% improvement in Assessment of SpondyloArthritis international Society response, *ASAS40* ≥ 40% improvement in Assessment of SpondyloArthritis international Society response, *ASDAS* Ankylosing Spondylitis Disease Activity Score, *ASQoL* Ankylosing Spondylitis Quality of Life, *BASDAI50* ≥ 50% improvement in Bath Ankylosing Spondylitis Disease Activity Index, *BASFI* Bath Ankylosing Spondylitis Functional Index, *BASMI* Bath Ankylosing Spondylitis Metrology Index, *bDMARD* biologic disease-modifying antirheumatic drug, *FACIT-F* Functional Assessment of Chronic Illness Therapy—Fatigue, *HI* Health Index, *ID* inactive disease, *IR* inadequate response, *LDA* low disease activity, *MASES* Maastricht Ankylosing Spondylitis Enthesitis Score, *MI* multiple imputation, *MMRM* mixed-effects model repeated measures, *NRI* non-responder imputation, *NRS* numeric rating scale, *PR* partial remission, *QD* once daily, *SD* standard deviation

**Supplementary Table 4.** Efficacy results at week 52 in prior TNFi/IL-17i subgroups

|  | **TNFi exposed^a^** | | **IL-17i exposed^a^** | |
| --- | --- | --- | --- | --- |
|  | **Placebo → upadacitinib 15 mg QD**  ***n=*183 / 168** | **Continuous upadacitinib 15 mg QD**  ***n=*181 / 165** | **Placebo → upadacitinib 15 mg QD**  ***n=*36 / 34** | **Continuous upadacitinib 15 mg QD**  ***n=*38 / 36** |
| **Patients, %** | **NRI-MI / AO** | **NRI-MI / AO** | **NRI-MI / AO** | **NRI-MI / AO** |
| ASAS40 response | 63.9 / 73.2 | 64.1 / 72.7 | 69.4 / 76.5 | 71.1 / 75.0 |
| ASAS20 response | 77.6 / 88.7 | 75.7 / 87.3 | 75.0 / 91.2 | 81.6 / 91.7 |
| ASAS PR | 29.5 / 32.7 | 30.4 / 33.9 | 19.4 / 20.6 | 26.3 / 27.8 |
| BASDAI50 | 54.6 / 62.5 | 56.9 / 63.6 | 55.6 / 58.8 | 63.2 / 66.7 |
| ASDAS ID | 27.0 / 30.1^b^ | 25.8 / 30.6^c^ | 9.3 / 9.4^d^ | 23.9 / 26.5^e^ |
| ASDAS LDA | 56.2 / 63.2^b^ | 55.2 / 64.3^c^ | 55.0 / 62.5^d^ | 61.1 / 67.6^e^ |
| **Mean change from baseline**^f^ | **MMRM / AO (SD)** | **MMRM / AO (SD)** | **MMRM / AO (SD)** | **MMRM / AO (SD)** |
| ASDAS | -1.97 / -2.00 (0.98)^b^ | -1.96 / -1.98 (1.06)^c^ | -1.75 / -1.73 (0.83)^d^ | -2.06 / -2.04 (1.04)^e^ |
| Total back pain^g^ | -4.35 / -4.40 (2.17) | -4.43 / -4.52 (2.32) | -4.13 / -4.16 (2.16) | -4.60 / -4.67 (2.45) |
| Nocturnal back pain^g^ | -4.39^h^ / -4.47 (2.14)^i^ | -4.44 / -4.48 (2.58) | -4.36 / -4.49 (2.13) | -4.69 / -4.58 (2.67) |
| Duration of morning stiffness^g^ | -4.26 / -4.26 (2.53) | -3.98 / -4.08 (3.13) | -3.15 / -3.04 (1.93) | -3.37 / -3.69 (3.11) |
| Severity of morning stiffness^g^ | -4.59 / -4.70 (2.13) | -4.45 / -4.47 (2.62) | -4.05 / -4.19 (2.26) | -4.21 / -4.28 (2.50) |
| Fatigue/tiredness^g^ | -3.63 / -3.65 (2.40) | -3.80 / -3.89 (2.46) | -3.19 / -3.28 (2.16) | -4.06 / -4.17 (2.89) |
| FACIT-F | 10.94^j^ / 10.80 (9.00)^k^ | 12.17^l^ / 12.34 (9.34)^b^ | 10.53^m^ / 10.56 (7.78)^n^ | 13.61^o^ / 13.96 (8.40)^m^ |
| BASFI | -3.46 / -3.51 (2.04) | -3.54 / -3.60 (2.34) | -3.64 / -3.70 (2.05) | -3.69 / -3.82 (2.52) |
| ASQoL | -7.14^h^ / -7.18 (4.45)^i^ | -7.21^l^ / -7.38 (5.02) | -5.95 / -6.13 (4.52) | -6.78 / -6.86 (4.59) |
| ASAS HI | -4.28^h^ / -4.22 (3.98) | -4.55 / -4.69 (3.92)^i^ | -3.75 / -3.76 (3.85)^m^ | -5.02 / -5.14 (3.97) |
| BASMI | -0.78^j^ / -0.79 (0.88)^p^ | -0.85^q^ / -0.83 (1.05)^r^ | -0.48 / -0.44 (0.78) | -0.83 / -0.86 (1.09)^m^ |
| MASES | -3.4^s^ / -3.2 (3.0)^t^ | -3.3^u^ / -3.6 (3.1)^v^ | -2.8^w^ / -2.8 (3.2)^x^ | -3.3^y^ / -3.5 (2.8)^y^ |

^a^Includes 19 patients with both prior TNFi and IL-17i exposure. ^b^*n=*163. ^c^*n=*157. ^d^*n=*32. ^e^*n=*34. ^f^Increasing negative values indicate improvement, apart from FACIT-F in which increasing positive values indicate improvement. ^g^NRS score 0–10. ^h^*n=*182. ^i^*n=*167. ^j^*n=*179. ^k^*n=*164. ^l^*n=*180. ^m^*n=*35. ^n^*n=*33. ^o^*n=*37. ^p^*n=*166. ^q^*n=*176. ^r^*n=*162. ^s^*n=*140. ^t^*n=*128. ^u^*n=*132. ^v^*n=*120. ^w^*n=*31. ^x^*n=*28. ^y^*n=*21

*AO* as observed, *ASAS* Assessment of SpondyloArthritis international Society, *ASAS20* ≥ 20% improvement in Assessment of SpondyloArthritis international Society response, *ASAS40* ≥ 40% improvement in Assessment of SpondyloArthritis international Society response, *ASDAS* Ankylosing Spondylitis Disease Activity Score, *ASQoL* Ankylosing Spondylitis Quality of Life, *BASDAI50* ≥ 50% improvement in Bath Ankylosing Spondylitis Disease Activity Index, *BASFI* Bath Ankylosing Spondylitis Functional Index, *BASMI* Bath Ankylosing Spondylitis Metrology Index, *FACIT-F* Functional Assessment of Chronic Illness Therapy—Fatigue, *HI* Health Index, *ID* inactive disease, *IL-17i* interleukin-17 inhibitor, *IR* inadequate response, *LDA* low disease activity, *MASES* Maastricht Ankylosing Spondylitis Enthesitis Score, *MI* multiple imputation, *MMRM* mixed-effects model repeated measures, *NRI* non-responder imputation, *NRS* numeric rating scale, *PR* partial remission, *QD* once daily, *SD* standard deviation, *TNFi* tumor necrosis factor inhibitor

**Supplementary Table 5.** Grade 3 and 4 laboratory parameters in the safety population

| **Parameter, n (%)** | **Any upadacitinib 15 mg QD**  **(*n=*414)** |
| --- | --- |
| Hemoglobin |  |
| Grade 3 (< 80 g/L) | 1 (0.3) |
| Lymphocytes |  |
| Grade 3 (0.2–< 0.5 × 10^9^/L) | 3 (0.7) |
| Grade 4 (< 0.2 × 10^9^/L) | 0 |
| Neutrophils |  |
| Grade 3 (0.5–< 1.0 × 10^9^/L) | 7 (1.7) |
| Grade 4 (< 0.5 × 10^9^/L) | 0 |
| ALT |  |
| Grade 3 (> 5–20 × ULN) | 3 (0.8) |
| Grade 4 (> 20 × ULN) | 0 |
| AST |  |
| Grade 3 (> 5–20 × ULN) | 5 (1.3) |
| Grade 4 (> 20 × ULN) | 0 |
| Creatinine |  |
| Grade 3 (> 3–6 × ULN or > 3 × baseline) | 1 (0.2) |
| Grade 4 (> 6 × ULN) | 0 |

*ALT* alanine aminotransferase, *AST* aspartate aminotransferase, *QD* once daily, *ULN* upper limit of normal
